# Maternal Parvovirus B19 Infection Causing First-Trimester Increased Nuchal Translucency and Fetal Hydrops

**DOI:** 10.1155/2019/3259760

**Published:** 2019-07-07

**Authors:** Olivia Grubman, Farrah Naz Hussain, Zoe Nelson, Lois Brustman

**Affiliations:** Department of Obstetrics and Gynecology, Mount Sinai West, Icahn School of Medicine at Mount Sinai, New York, NY 10019, USA

## Abstract

This is a case report of a 31-year-old primigravida who was diagnosed with an asymptomatic acute parvovirus B19 infection in the second trimester of pregnancy and its suspected association with an increased nuchal translucency (NT) measurement. Parvovirus B19 is a single-stranded DNA virus that is cytotoxic to erythroid progenitor cells, causing inhibition of erythropoiesis. While maternal disease is usually mild, fetal infection can result in spontaneous abortion, aplastic anemia, nonimmune fetal hydrops, and fetal demise. This fetus had an increased NT of 3.2 mm at 11 weeks' gestation with a normal male karyotype and microarray analysis on chorionic villi sampling, in addition to a normal fetal echocardiogram at 15 weeks' gestation. The anatomy scan at 20 weeks' and 1-day gestation revealed fetal ascites, pleural effusion, and increased middle cerebral artery peak systolic velocity suspicious for fetal anemia. At this time, maternal serology for parvovirus was positive for IgM and IgG. Amniocentesis, cordocentesis, and intrauterine transfusion were performed. The amniocentesis revealed elevated parvovirus B19 DNA, quantitative PCR (2,589,801 copies/mL, reference range <100 copies/mL). The patient delivered a viable male fetus at 37 weeks' and 6-day gestation, without sequelae of the previously noted hydrops. Parvovirus B19 infection should be a consideration when evaluating increased NT and hydrops fetalis. It warrants close antepartum surveillance and possible intrauterine fetal transfusions. With prompt recognition, proper treatment, and surveillance, these patients can go on to achieve healthy term deliveries. Long-term outcomes of delivered infants require further study.

## 1. Case Presentation

**Figure 1:**
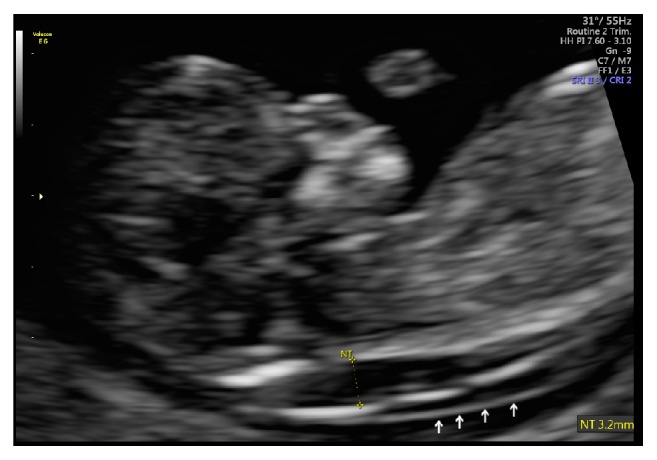
Nuchal translucency 3.2 mm.

A 31-year-old primigravida was referred to our Maternal Fetal Medicine practice after a routine first-trimester ultrasound at 11 weeks' gestation that revealed a nuchal translucency (NT) of 3.2 mm (Figure 1). The crown rump length measured 11 weeks two days, which was consistent with the patient's last menstrual period which placed her gestational age at 11 weeks. A chorionic villi sample (CVS) was performed at that time. Cytogenetic analysis from the chorionic villi showed a normal male karyotype of 46,XY. The results of the fluorescent in situ hybridization (FISH analysis), Noonan's panel, and microarray for aneuploidy testing were normal. Due to the elevated NT, the patient was referred for a fetal echocardiogram at 15 weeks and 5 days' gestation which revealed a regular atrial rhythm with no structural cardiac abnormalities. An early anatomy ultrasound at 16 weeks and 0 days' gestation was normal, with fetal size appropriate for gestational age and no evidence fetal anomalies.

**Figure 2:**
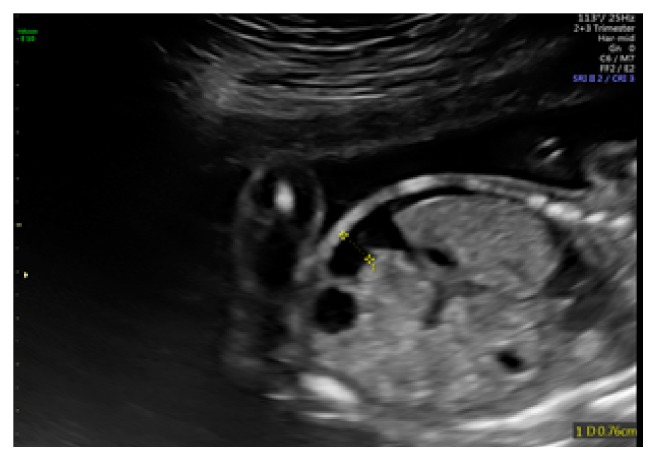
Anatomy ultrasound at 20 weeks' and 1-day gestation with fetal ascites.

**Figure 3:**
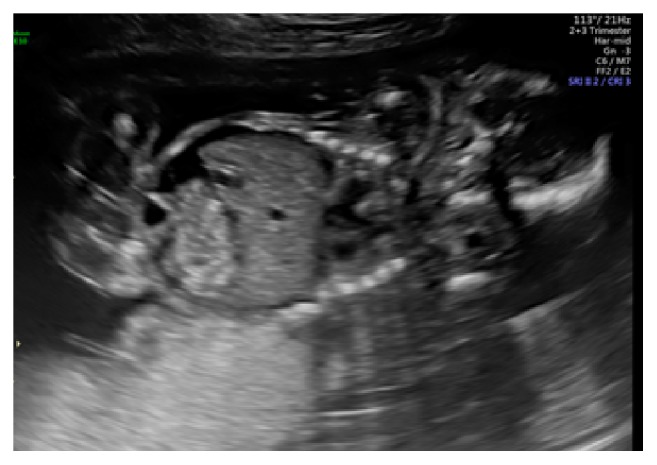
Anatomy ultrasound at 20 weeks' and 1-day gestation with fetal ascites.

**Figure 4:**
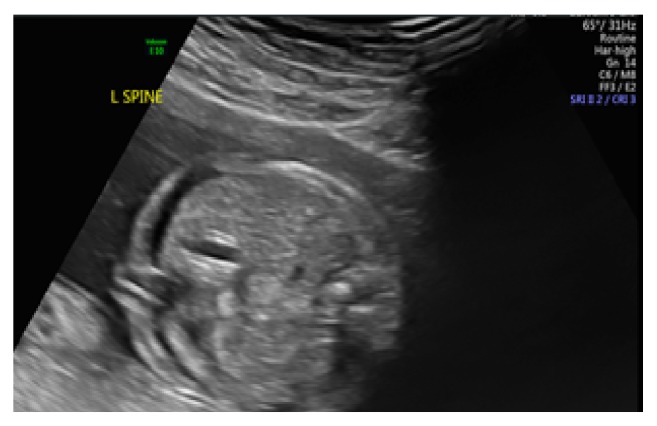
Anatomy ultrasound at 20 weeks' and 1-day gestation with fetal ascites.

A comprehensive anatomy scan at 20 weeks' and 1-day gestation revealed fetal ascites and a small pleural effusion (Figures 2, 3, and 4). No pericardial effusion was observed. The middle cerebral artery peak systolic velocity (MCA PSV) was elevated (1.95 multiples of the median (MoM)), which was suggestive of fetal anemia. At this time, routine serologic testing for herpes simplex virus (HSV), parvovirus, toxoplasmosis, and cytomegalovirus was performed. The patient underwent amniocentesis with polymerase chain reaction (PCR) testing for parvovirus, toxoplasmosis, cytomegalovirus (CMV), and adenovirus.

A follow-up fetal echocardiogram showed no structural cardiac abnormalities or rhythm abnormalities seen as a cause of hydrops. However, the fetus was noted to have scalp edema, worsening pleural effusion and ascites, and, now, a pericardial effusion. Fetal hemodynamics was normal, including Doppler flow patterns in the umbilical artery and vein, ductus venosus and arteriosus, and middle cerebral artery. Fetal ascites was still present at 20 weeks and 2 days' gestation. The MCA PSV was elevated at 75 cm/second. Maternal parvovirus B19 IgG and IgM were elevated, with values of 5.3 and 2.4 (negative or equivocal <1.1), respectively. At this time, the patient recalled an upper respiratory illness at approximately 9 to 10 weeks' gestation. She denied rash, arthralgias, or knowledge of exposure to a child or an adult with symptoms consistent with a parvovirus infection. Given the above findings, the decision was made that day to perform an amniocentesis, a cordocentesis, and an intrauterine transfusion based on the presumptive diagnosis of fetal anemia secondary to an acute parvovirus infection. The fetal hematocrit was 10%, indicating anemia, and 12 cc of blood was transfused, raising the fetal hematocrit to 25%. At the end of the procedure, the MCA PSV was 45 cm/sec, with decreased systolic/diastolic (S/D) ratio of approximately 2.0.

At 20 weeks and 5 days' gestation, the results of the amniocentesis demonstrated elevated parvovirus B19 DNA, quantitative PCR (2,589,801 copies/mL, reference range <100 copies/mL). Fetal ascites persisted the following day at 20 weeks and 6 days' gestation. The MCA PSV was 42 cm/min. A cordocentesis revealed a fetal hematocrit of 24%, and an additional intrauterine transfusion was given for a total volume of 15 cc. The following week, there was no evidence of fetal ascites and the MCA PSV was normal. Subsequent maternal serologic testing for herpes simplex, cytomegalic, and toxoplasmosis virus was negative.

For the remainder of the second trimester, ultrasounds were performed every other week to assess fetal growth and MCA Doppler flow. Weekly antenatal surveillance was performed in the third trimester and was consistently reassuring.

At 37 weeks and 5 days' gestation, the patient was induced for gestational hypertension. She delivered a viable male fetus weighing 2880g at 37 weeks and 6 days. The neonatal Apgar scores were 8 and 9 at 1 and 5 minutes, respectively. At the time of this report, the infant is doing well and developing appropriately for 5 months of age.

## 2. Discussion

Our case report is one of few in the literature that suggests an association of an increased NT measurement with fetal anemia due to an asymptomatic parvovirus B19 infection in the mother. The fetal anemia was successfully treated, when diagnosed in second trimester, with fetal blood transfusions and subsequent prolongation of the pregnancy to term with no apparent sequelae.

Human parvovirus B19 is a small, nonenveloped, single-stranded DNA virus. It is disseminated by respiratory secretions or from hand to mouth contact. Other modes of transmission include transfusion of blood products and transplacental transfer. Also known as erythema infectiosum, parvovirus is a common childhood illness that is recognized by the classic bright facial rash or “slapped cheek rash.” The virus generally affects children from age four to eleven. In addition to the facial rash, there is commonly a lacelike rash on the extremities and trunk. Conversely, adults generally do not present with a rash. Arthropathies are the most common symptom in adults, affecting up to 50% of pregnant women with acute B19 infection. Arthralgia including the hands, wrists, knees, and ankles can occur, lasting for one to two weeks [1]. It has been reported that 50-70% of pregnant women are immune to parvovirus B19, with the majority having no recollection of when they were infected. As with our patient, if infected with parvovirus in pregnancy, up to 70% of women will be asymptomatic.

The incidence of acute B19 infection in pregnancy is 3.3% to 3.8% [2, 3], with a transplacental transmission rate of approximately 30% [4]. Parvovirus infection that occurs during the first trimester has been associated with a 71% increased risk of fetal loss [5]. The fetal demise rate is up to 15% early in the second trimester and prior to 20 weeks. After 20 weeks, the rate of fetal demise decreases to about 2.3%, as the viral insult to the bone marrow is not as detrimental [2, 5]. It has been reported that fetal demise can occur up to 12 weeks after maternal infection in any trimester [6].

Parvovirus B19 replicates in red blood cell (RBC) precursors mainly in the bone marrow and fetal liver, which can result in hemolysis and RBC aplasia. Aplastic crisis in a fetus can lead to cardiac failure, nonimmune hydrops, and death. Parvovirus infects primarily the erythroid cell line and therefore can cause fetal anemia which can lead to cardiac failure with hydrops fetalis [7]. It is estimated that parvovirus accounts for 8% to 10% of nonimmune hydrops [2]. The suspected mechanism for the development of nonimmune hydrops fetalis involves the infection of fetal erythroid precursor cells with parvovirus, with subsequent erythroid hypoplasia. This then leads to severe fetal anemia, tissue hypoxia, and, eventually, a compensatory increase in cardiac output followed by heart failure [8]. Furthermore, it has been proposed that fetal death could also be due to myocarditis causing cardiac failure as a result of the affinity of the virus for cardiac tissue [9–11]. It has also been reported that the parvovirus B19 is toxic to the megakaryocytes, hepatocytes, and placental cells which could also attribute to a fetal demise [12].

There are few reports in the literature of increased NT associated with parvovirus [7, 12, 13]. Although an increased NT is not commonly associated with fetal infection, parvovirus B19 is the only infection that has a reported association with an increased NT [12]. Markenson et al. [14] reported a case in which they found an increased NT (3.7 mm) in a fetus with a normal karyotype, but parvovirus was detected in the amniotic fluid. In this case, the patient recalled that she was exposed to a child with a high fever and rash around 5 to 6 weeks' gestation. She went on to a delivery of a viable term male infant with redundant nuchal skin thickening. Another case found an NT (4.2 mm) and reversed a-wave in the ductus venosus (DV) during a first-trimester ultrasound. As with the prior case, the mother reported contact with children with erythema infectiosum and had positive serologic testing (IgG and IgM antibodies) for parvovirus B19. Unfortunately, in this case, a fetal demise occurred two weeks after diagnosis with parvovirus infection confirmed in the fetus based on immunocytochemical studies using specific antibodies [12]. Finally, Kempe et al. [7] report two cases with an increased NT, elevated MCA PSV, and cardiomegaly, all suspicious for fetal anemia in the first trimester. Maternal parvovirus infection was confirmed with a positive PCR. In each case, the fetus received a 3-mL umbilical vein transfusion of packed red blood cells. In one of the cases, three more intrauterine blood transfusions were necessary until the fetal hydrops resolved. There were good neonatal outcomes in both cases.

Based on the case reports above, although rare, acute parvovirus infection should be considered in the differential diagnosis, as an etiology for an increased NT. An early diagnosis can enable intervention via intrauterine blood transfusions, which can potentially cure hydrops fetalis. In our case, at the time of the finding of an increased NT in first trimester, the diagnosis of parvovirus infection was not considered and the patient's subtle history of upper respiratory symptoms was missed. If we had considered parvovirus as an etiology for an increased NT, the diagnosis could have been made earlier and, thus, the fetus could have been followed more closely with serial sonograms and Doppler studies.

As we have described, the management of fetuses with hydrops or anemia due to parvovirus B19 infection includes cordocentesis to assess fetal hemoglobin and reticulocyte count, and intrauterine fetal transfusion, if necessary. Furthermore, if the fetus is at or near term, delivery should be considered. A summary of 14 studies including 1436 cases of fetal parvovirus infection found a fetal survival rate of 82% with transfusion compared to a fetal survival rate of 55% for those who were not transfused [2]. Two to three transfusions may be required before resolution of the fetal hydrops or anemia, which usually takes place three to six weeks after the first transfusion. Recommended monitoring includes weekly ultrasonography and Doppler assessment to assess the MCA, which is a noninvasive method to measure a fetus at risk for anemia [6, 15, 16].

Several small studies have examined long-term outcomes after intrauterine transfusion for parvovirus B19 infection during pregnancy. These studies, ranging from two to 28 patients, found rates of minor neurodevelopmental impairment from 0 to 18.8% and rates of major neurodevelopmental impairment from 0 to 12.5%. Neurological abnormalities included mild developmental delay and more severe abnormalities such as ataxia and hypertonia. The causes for increased rates of cerebral injury and adverse neurodevelopmental outcomes in antenatal parvovirus B19 are poorly understood. Possible mechanisms include compromised fetal status due to severe anemia and hydrops or adverse reaction related to the virus itself. It is clear that further long-term studies are needed on children who have previously undergone intrauterine fetal transfusion for hydrops related to parvovirus [17–22]. Given this data, patients should be counseled thoroughly on the risks and benefits of intrauterine transfusion for the “cure” of the effects of parvovirus in the fetus, as well as possible long-term sequelae. This should be a shared decision making process with the patient and the physician.

This case, as well as others in the literature, emphasizes parvovirus in the differential of an elevated fetal NT. Parvovirus is the only infectious cause of an increased fetal NT and should be a consideration in these patients along with chromosomal and cardiac abnormalities. Early recognition provides the opportunity for early management of fetal hydrops and increased fetal survival rates. However, long-term sequelae of antenatal parvovirus and intrauterine transfusions on the infant, including neurodevelopmental impairment, are poorly understood and require further study. The case described, as well as others, provides information relevant for patient counseling and shared decision-making.

## 3. Conclusion

It is our recommendation that parvovirus should be considered in the differential of an increased NT in first trimester. Maternal serology with IgM and IgG specific antibody titers should be performed. A patient's history should be elicited, but this may be noncontributory based on the fact that the majority of parvovirus infections in pregnancy are asymptomatic.
